# Enhancing subthreshold slope and ON-current in a simple iTFET with overlapping gate on source-contact, drain Schottky contact, and intrinsic SiGe-pocket

**DOI:** 10.1186/s11671-023-03904-7

**Published:** 2023-09-29

**Authors:** Jyi-Tsong Lin, Kuan-Pin Lin, Kai-Ming Cheng

**Affiliations:** Department of Electrical Engineering, National Sun Yat-Sen University, Kaohsiung, 80424 Taiwan, R.O.C.

**Keywords:** TFET, Line-tunneling, Schottky contact, Without ion implantation, Low subthreshold swing

## Abstract

In this paper, we present a new novel simple iTFET with overlapping gate on source-contact (SGO), Drain Schottky Contact, and intrinsic SiGe pocket (Pocket-SGO iTFET). The aim is to achieve steep subthreshold swing (*S.S*) and high *I*_ON_ current. By optimizing the gate and source-contact overlap, the tunneling efficiency is significantly enhanced, while the ambipolar effect is suppressed. Additionally, using a Schottky contact at the drain/source, instead of ion implantation drain/source, reduces leakage current and thermal budget. Moreover, the tunneling region is replaced by an intrinsic SiGe pocket posing a narrower bandgap, which increases the probability of band-to-band tunneling and enhances the *I*_ON_ current. Our simulations are based on the feasibility of the actual process, thorough Sentaurus TCAD simulations demonstrate that the Pocket-SGO iTFET exhibits an average and minimum subthreshold swing of *S.S*_avg_ = 16.2 mV/Dec and *S.S*_min_ = 4.62 mV/Dec, respectively. At *V*_D_ = 0.2 V, the *I*_ON_ current is 1.81 $\times$ 10^–6^ A/μm, and the *I*_ON_/*I*_OFF_ ratio is 1.34 $\times$ 10^9^. The Pocket-SGO iTFET design shows great potential for ultra-low-power devices that are required for the Internet of Things (IoT) and AI applications.

## Introduction

The demand for low-power devices suitable for high-energy-efficient applications like the Internet of Things (IoT) and artificial intelligence (AI) has grown significantly, as outlined in the International Roadmap for Devices and Systems (IRDS) [1]. However, conventional complementary metal–oxide–semiconductor field-effect transistors (MOSFETs) are constrained by the Boltzmann limit [2], which restricts their ability to achieve low power consumption due to a minimum subthreshold swing of 60 mV/Dec at room temperature.

To overcome this limitation, novel device types have been explored, including ferroelectric FETs [3] and tunnel FETs (TFETs) [4]. Among these, TFETs utilize minority carrier band-to-band tunneling (BTBT) to achieve subthreshold swings below 60 mV/Dec at room temperature [5, 6]. However, TFETs face challenges like low* I*_ON_ current, limited *I*_ON_/*I*_OFF_ current ratios, and gate-bias and trap dependent subthreshold swing (S.S) degradation. Gate bias conditions can lead to undesirable ambipolar and trap-assisted tunneling (TAT) effects [7]. Addressing the low ON current, previous studies explored strategies such as heterojunctions and heavy pocket doping to enhance conduction current [8–12]. However, these methods involve intricate fabrication processes, making TFETs less competitive in the semiconductor landscape, as evidenced by their absence from the mainstream technologies listed in the 2018 IRDS [13].

In the quest to develop practical, high-performance, low-power TFETs, we recognize the limitations of existing approaches. While some studies have investigated line tunneling mechanisms to simplify manufacturing and increase conduction current [14–17], precise ion implantation in miniature devices remains a challenge. Alternative strategies, such as charge plasma [18–20] and advanced gate configurations [21, 22], aimed to simplify the doping process and boost on-current but have not provided a complete solution. Noteworthy, some alternative works try to enhance line tunneling using symmetry structure. Such as, A. Anam presented an undoped vertical dual-bilayer TFET [23], emphasizing the complexity of the fabrication process and the trade-offs between source and drain scaling. The study highlighted the challenges posed by symmetric structures, which can exacerbate ambipolar effects. X. Jin explored a symmetry high-low–high Schottky barrier-based bidirectional TFET [24]. Their work emphasized the impact of gate-source line tunneling on device performance. It highlights the trade-offs between symmetry and ambipolar effects. In essence, designing a TFET with a symmetric structure that utilizes more line tunneling for improved device performance is impossible when starting with the traditional gated-PIN TFET, which inherently has an asymmetric design. While efforts in the source engineering to maximize line tunneling can indeed increase *I*_ON_ (on current), attempting to apply the same source engineering principles to a symmetric drain structure results in severe and uncontrollable drain leakage current and ambipolar effects. Furthermore, as it remains based on the conventional PIN TFET architecture, it is still susceptible to point tunneling, which is sensitive to energy traps and cannot be avoided.

In light of these challenges, we propose a novel Pocket-SGO iTFET design that replaces ion implantation with Schottky contacts, offering a streamlined and efficient manufacturing process. While previous studies have investigated Schottky barrier TFETs (SBTFETs) [25–28], they often involve complex fabrication steps. Our approach aims to overcome these limitations and provide a practical solution for achieving high performance, low-power TFETs.

**Fig. 1:**
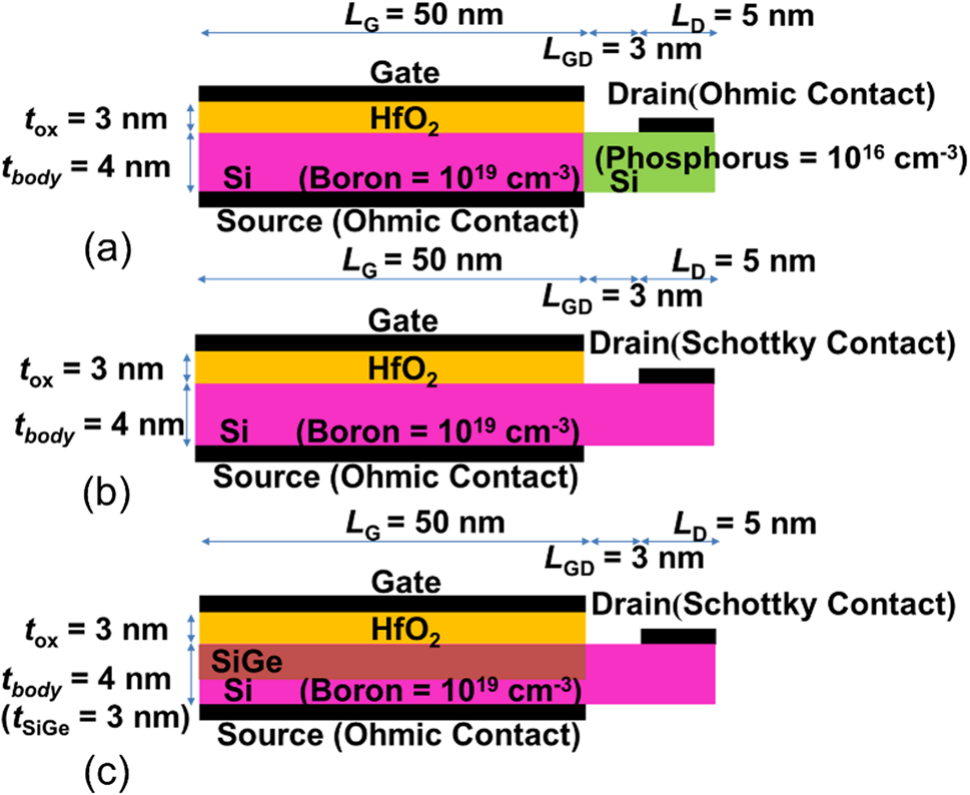
Schematics of **a** PN-SGO TFET with ion implantation drain, **b** SGO iTFET with Schottky contact drain, and **c** Pocket-SGO iTFET with Schottky contact drain and SiGe pocket

In this paper, acknowledging these challenges, we introduce a novel class of TFETs: (a) PN-SGO TFET with ion implantation drain, (b) SGO iTFET with Schottky contact drain, and (c) Pocket-SGO iTFET with Schottky contact drain and SiGe pocket, as illustrated in Fig. 1. These designs tackle the issues of low ON current, high cost, and the prevalent TAT and ambipolar effects observed in conventional PIN TFETs. The incorporation of overlapped gate-source contact structures facilitates large-area band-to-band tunneling with increased band-to-band generation rates, leading to augmented conduction current. Furthermore, the comprehensive utilization of line tunneling effectively mitigates ambipolar and TAT effects, common issues encountered in traditional PIN TFETs relying on point tunneling. By incorporating a Schottky contact at the drain, leakage current is reduced, and ambipolar behavior is alleviated. Substituting the P–N junction with a metal–semiconductor junction reduces ion implantation steps, subsequently lowering thermal budgets and total costs. The introduction of a SiGe pocket enhances on-current and reduces subthreshold swing by utilizing a narrower bandgap intrinsic semiconductor. These innovative structures require no implantation and annealing technologies and employ a straightforward manufacturing process, significantly reducing the thermal budget. Our results demonstrate that the Pocket iTFET exhibits high *I*_ON_, a substantial *I*_ON_/*I*_OFF_ ratio, low *S.S*, and minimal leakage current while effectively eliminating ambipolar and TAT effects. This makes it highly suitable for ultra-low-power applications.

## Device structure and operating mechanism

**Fig. 2:**
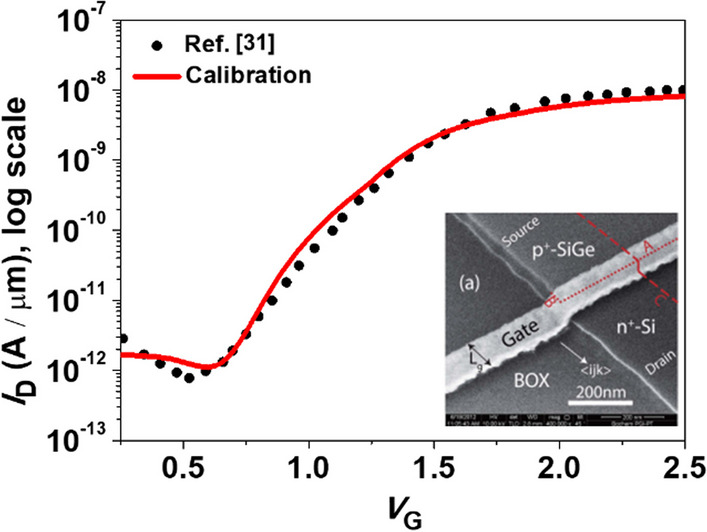
TCAD model calibration using experimental data

Our simulations using Sentaurus TCAD [29], which include models for Shockley–Read–Hall recombination, bandgap narrowing, doping dependence, nonlocal band-to-band tunneling, high-field dependence of mobility, trap-assisted tunneling. Due to the thin channel thickness, it is necessary to consider the quantum confinement effect [30]. To ensure the accuracy and feasibility of our simulations, we have calibrated our simulation model using an actual fabricated TFET [31], as shown in Fig. 2. Equation (1) represents the calculation formula for tunneling generation rate, in which the related *A*_BTBT_, *A*_BTBT_, *P* and ${F}_{O}$ are carefully calibrated. To accurately simulate the effects of TAT (trap-assisted tunneling), we employed the Hurkx TAT Model within the framework of the SRH (Shockley–Read–Hall) generation-recombination model. We also carefully fine-tuned the sole parameter of this model, which is the carrier tunneling mass (*m*_t_). For SiGe, the extracted values of electron and hole *m*_t_ were 0.15*m*_e_ (electron mass) and 0.51 *m*_e_ respectively, which can be derived by Linear Interpolation from those of Silicon and Germanium. All the parameter adjustments made during the calibration process are summarized in Table 1.

1 $$G = A_{BTBT} \left( {\frac{F}{{F_{O} }}} \right)^{P} {\text{exp}}\left( { - \frac{{B_{BTBT} }}{F}} \right)$$

**Table 1:** Calibration parameters

Parameters	Si_x_Ge_1-x_ (x = 1)	Si_x_Ge_1-x_(x = 0)
*µ*_n_	1414 m^2^/(V^.^s)	3900m^2^/(V^.^s)
*µ*_p_	470 m^2^/(V^.^s)	1900m^2^/(V^.^s)
*A*_BTBT_	4 × 10^14^ cm^−3^ s^−1^	9.1 × 10^16^ cm^−3^ s^−1^
*B*_BTBT_	1.9 × 10^7^ V^.^cm^−1^	4.9 × 10^6^ V^.^cm^−1^
*P*	2.5	2.5
*F*_0_	1 V/cm	1 V/cm
Electron* m*_t_	0.20 *m*_e_	0. 13 *m*_e_
Hole* m*_t_	0.80 *m*_e_	0. 38 *m*_e_

The dimensions of the structures shown are provided in Table 2, with a gate length (*L*_G_) of 50 nm for this device. The length of the drain (*L*_D_) is 5 nm, and the drain-semiconductor junction is a Schottky contact. The length of the source (*L*_S_) is 50 nm, and the source-semiconductor junction is an ohmic contact. The gate-to-drain length (*L*_GD_) is 3 nm. The gate oxide (*t*_ox_) is made of HfO_2_ and has a thickness of 3 nm. The body (*t*_body_) is made of Si and has a thickness of 4 nm with heavy P-doping (10^19^ cm^−3^), while the Si_0.7_Ge_0.3_ pocket is intrinsic.

**Table 2:** Device parameters used for simulations

Device parameters	
Body thickness (*t*_body_)	4 nm
Gate dielectric thickness (*t*_ox_)	3 nm
Pocket thickness (*t*_SiGe_)	3 nm
Gate length (*L*_G_)	50 nm
Drain length (*L*_D_)	5 nm
Source length (*L*_S_)	50 nm
Gate to drain length (*L*_GD_)	3 nm

**Fig. 3:**
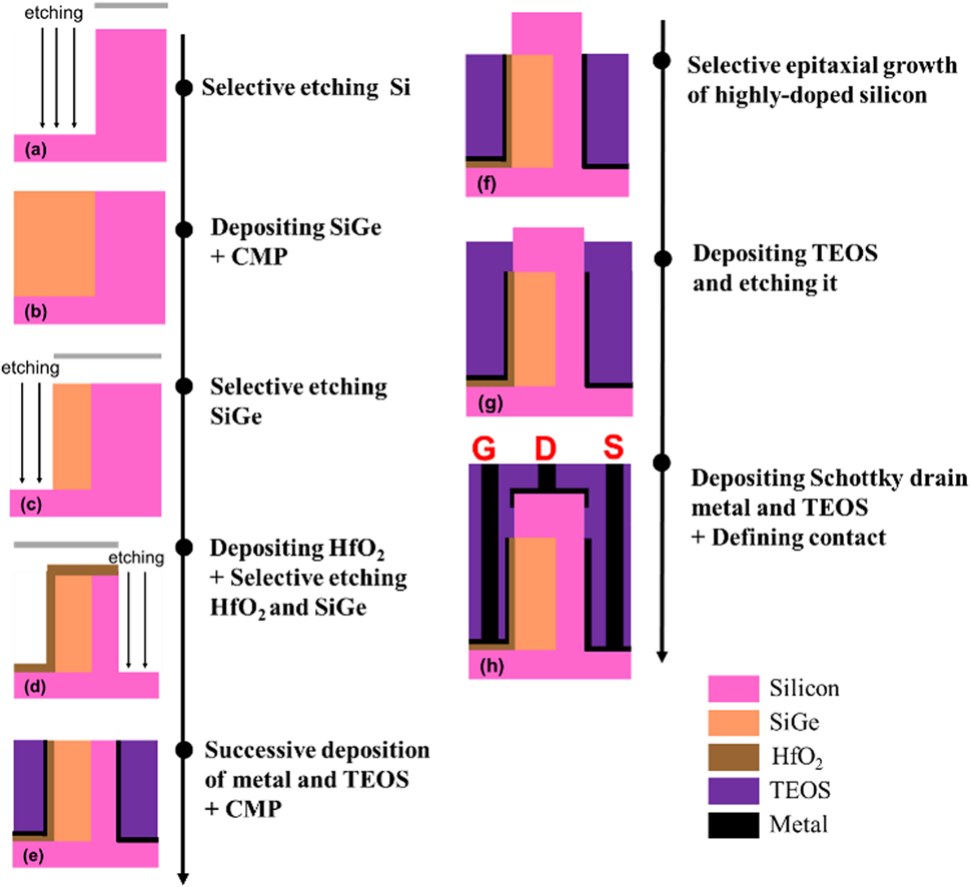
Simple process flow for Pocket-SGO iTFET

The manufacturing process involves several crucial steps. Firstly, in Fig. 3a, the silicon substrate is defined by using a photomask to create patterns. Next, in Fig. 3b, SiGe is epitaxial growth onto the substrate [32, 33], followed by chemical mechanical polishing (CMP) to achieve the desired structure. Subsequently, in Fig. 3c, the SiGe active area is defined. In the subsequent step, shown in Fig. 3d, HfO_2_ is deposited using atomic layer deposition (ALD), and then etched to define the desired patterns. Continuing, Fig. 3e demonstrates the consecutive deposition of metal and TEOS, followed by CMP to expose the Si and SiGe regions. Additionally, Fig. 3f explains the utilization of selective epitaxial growth process to grow heavily doped silicon in the exposed Si and SiGe regions. Furthermore, in Fig. 3g, TEOS is deposited and subsequently etched. Finally, in Fig. 3h, the deposition of metal with TEOS is shown, forming contact holes that allow for the subsequent creation of contacts.

**Fig. 4:**
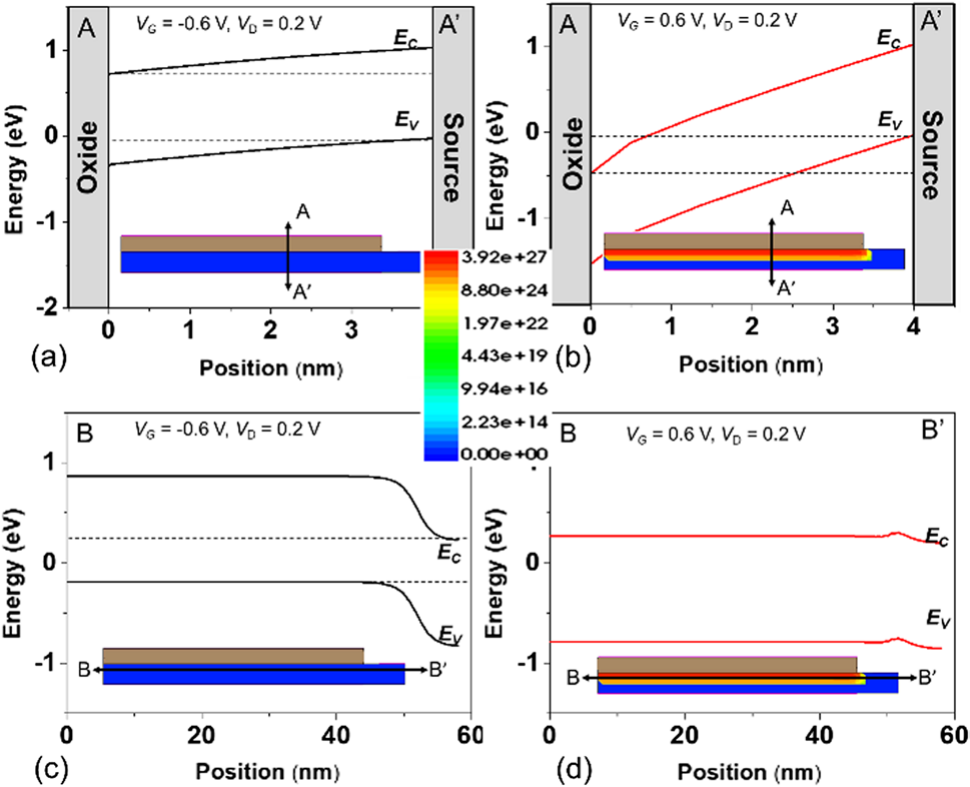
Electron band-to-band generation rate and energy bandgap diagram along the cutline *A–A*′ **a** ambipolar-state (*V*_G_ = − 0.6 V, *V*_D_ = 0.2 V), **b** on-state (*V*_G_ = 0.6 V, *V*_D_ = 0.2 V) and *B–B*′ **c** ambipolar-state (*V*_G_ = − 0.6 V, *V*_D_ = 0.2 V), **d** on-state (*V*_G_ = − 0.6 V, *V*_D_ = 0.2 V). The *I*_ON_ current generation mechanism is line-tunneling and there is no ambipolar effect at *V*_G_ < 0

**Fig. 5:**
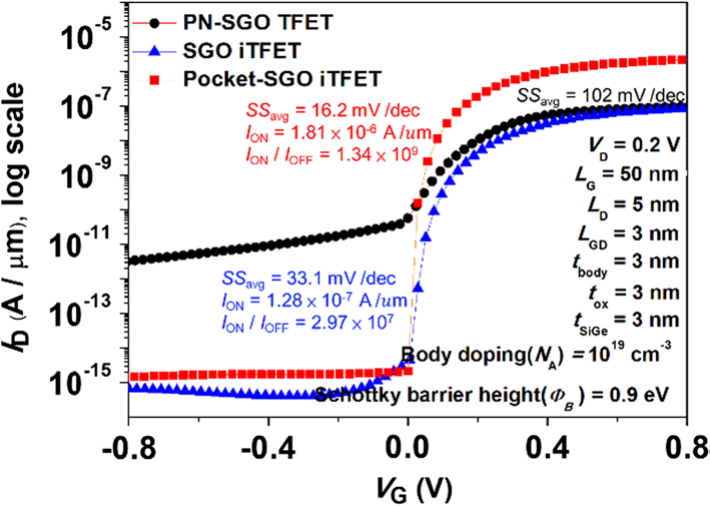
Simulated *I*_D_–*V*_G_ characteristics of our proposed TFETs

Figure 4 shows the electron band-to-band generation rate and energy bandgap diagrams (a) ambipolar-state (b) on-state along the cutline *A–A*′ for point-tunneling verification, and (c) ambipolar-state (d) on-state along the cutline *B–B*′ for line-tunneling. It is observed that the device exhibits mainly *I*_ON_ current mechanism dominated by line-tunneling, and there is no appearance of point tunneling. It can effectively suppress the ambipolar effect because there is no tunneling current when *V*_G_ is less than 0. Figure 5 shows the *I*_D_–*V*_G_ characteristics of our proposed TFET. As shown, the proposed structure exhibits higher ionic current in the on-state due to the tunneling mechanism involving full overlap of the source metal and gate regions, and no ambipolar effect when *V*_G_ is less than 0. At *V*_D_ = 0.20 V, the SGO iTFET exhibits an *I*_ON_ of 1.28 $\times$ 10^–7^ A/μm, a *S.S*_min_ of 9.85 mV/Dec, a *S.S*_avg_ of 31.9 mV/Dec, and an *I*_ON_/*I*_OFF_ ratio of 2.97 $\times$ 10^7^, while the Pocket iTFET exhibits an *I*_ON_ of 1.81 $\times$ 10^–6^ A/μm, *S.S*_min_ of 4.33 mV/Dec, *S.S*_avg_ of 16.2 mV/Dec, and *I*_ON_/*I*_OFF_ ratio of 1.34 $\times$ 10^9^.

## Result and discussion

For clarity, as shown in Fig. 1, the three novel simple inductive TFET (iTFET) has been designed for the ultra-low power supply. The iTFET unlike a conventional TFET being a Gated PIN diode, can be a Gated PPN diode or Gated PNN diode, in which the TFET structures are formed by induced region from Schottky Contact, and their dominated current transportation is line tunneling of minority carriers. The line-tunneling indeed occurs between the inductive inversion layer and substrate, or between the inversion layer and the layer induced by Schottky contact. That is why we call it as iTFET. In addition, it is the most worthwhile noting that the 0.2 V drain bias *V*_DS_ is employed for all the simulations and characterizations, which imply the lower power supply voltage can be employed even low to 0.2 V. The gate bias used can be more than 0.2 V in the simulations which allow us to cover and understand all the effects, such as ambipolar effects and threshold voltage shift phenomena.

### PN implantation drain and Schottky contact drain

**Fig. 6:**
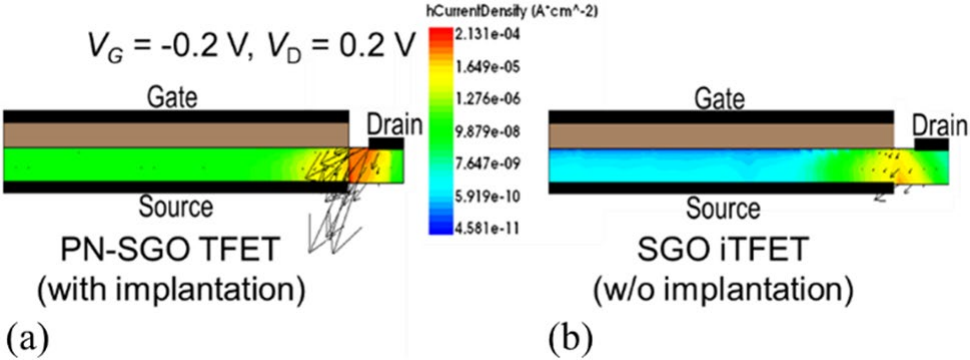
Hole current density and vector diagrams for TFETs (**a**) with implantation drain and (**b**) with Schottky contact drain respectively

In Fig. 5, the leakage current of SGO iTFET and Pocket-SGO iTFET is significantly lower compared to that of an implanted PN-SGO TFET. This difference is evident from the current densities and vectors shown in Fig. 6, where it is evident that the hole current of the SGO iTFET is significantly lower than that of the PN-SGO TFET when *V*_G_ =  − 0.2 V. Leakage currents can be attributed to the presence of Schottky barriers that block the flow of majority carriers from the metal to the semiconductor. Therefore, the leakage current is significantly reduced. In addition, the use of Schottky contact drain instead of ion implantation drain can greatly reduce the thermal budget.

### Doping consideration

**Fig. 7:**
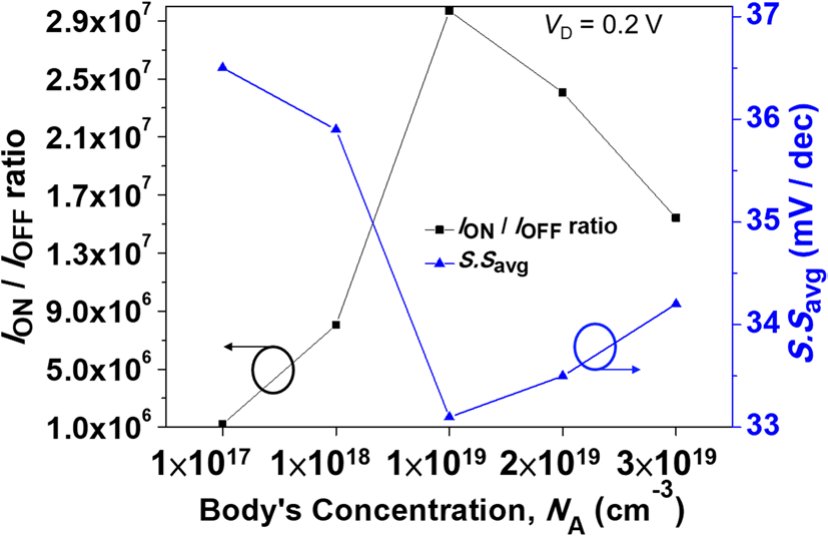
Simulated *I*_ON_/*I*_OFF_ ratio and *S.S*_avg_ for SGO iTFET with various body doping. The best performance is achieved at a concentration of 1 × 10^19^ cm^−3^

**Fig. 8:**
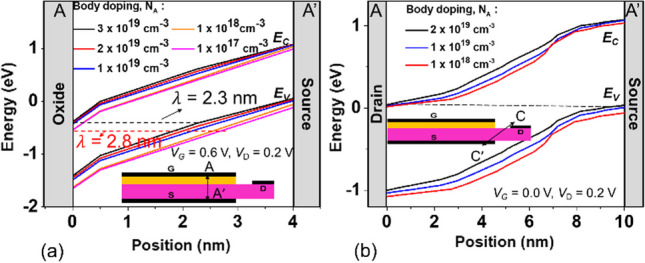
Energy bandgap diagram along the cutline **a** *A–A′* (b) *C–C′* for SGO iTFET. **a** The concentration increases, the tunneling distance becomes shorter. **b** Tunneling current occurs in the source and drain region when the concentration is greater than 10^19^ cm^−3^

The concentration of the body has a significant impact on the component, since the component we designed is a PN junction formed by the inversion layer controlled by the gate and the source region body with its own concentration, which produces line tunneling. Figure 7 shows the simulated *I*_ON_/*I*_OFF_ ratio and *S.S*_avg_ for SGO iTFET with various body doping. As shown, the best performance was achieved at a concentration of 10^19^ cm^−3^. As Fig. 8a energy band diagram of the SGO iTFET along the cutline *A-A’*, as the doping concentration increases, the tunneling distance of electrons from the conduction band to the valence band becomes shorter. Therefore, the probability of band-to-band tunneling increases, resulting in an increase in the *I*_ON_ current. Moreover, The *I*_ON_ current begins to decay and the *I*_OFF_ leakage current increases when the doping concentration exceeds 10^19^ cm^−3^. From Fig. 8b energy band diagram of the SGO iTFET along the cutline *C–C*′, it can be observed that band-to-band tunneling occurs between source and drain, which leads to performance degradation.

### Overlapping issue of gate on source metal

**Fig. 9:**
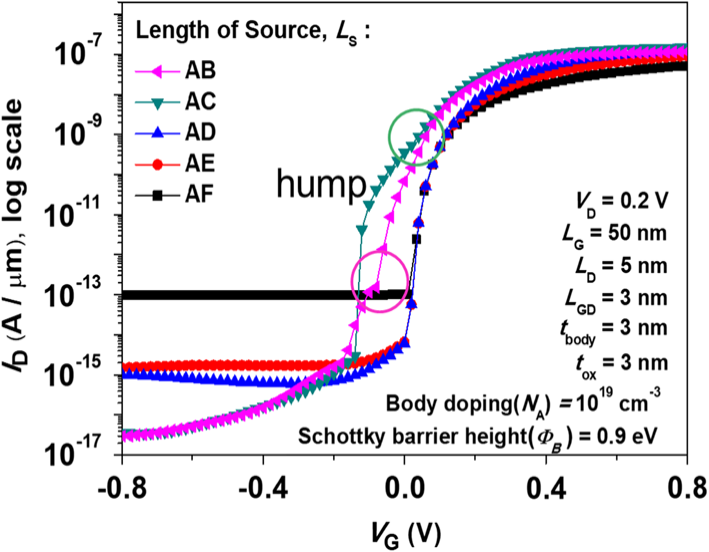
Simulated *I*_D_–*V*_G_ characteristics for iTFET with gate-source complete overlap or partial overlap. When there is complete gate-source overlap and the source length exceeds the gate length, the device performance will degrade. In the case of partial gate-source overlap, a hump effect occurs (as indicated by the circles), which can also have a negative impact on the device performance

**Fig. 10:**
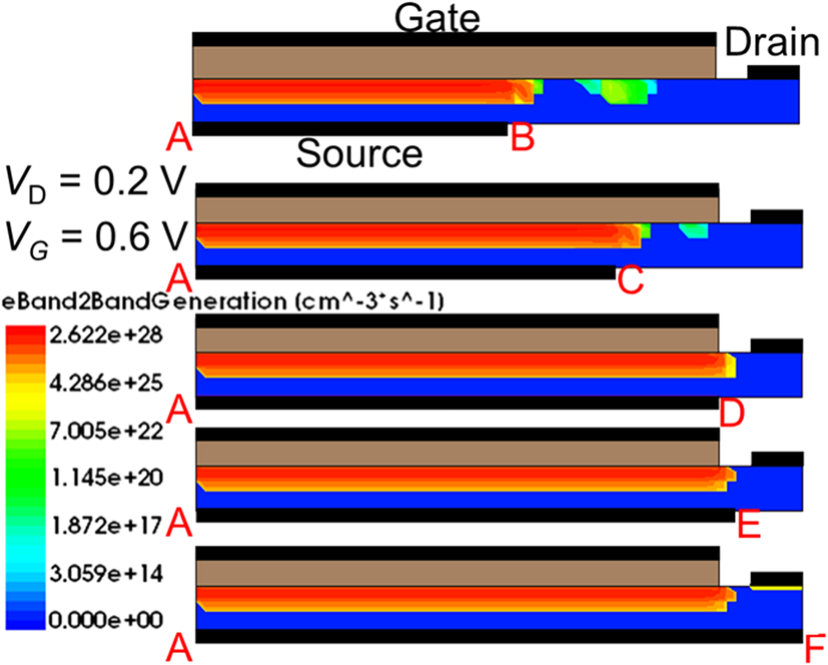
Electric band-to-band generation for SGO iTFET with gate-source complete overlap or partial overlap at on-state. The tunneling area varies with the overlap length between the gate and source, and reaches a maximum value at *L*_S_ = *L*_G_

The following section focuses on the impact of gate-source complete overlap or partial overlap on device performance. Figure 9 shows the simulated *I*_D_-*V*_G_ characteristics for SGO iTFET of gate-source complete overlap or partial overlap. The SGO iTFET exhibits optimal performance at *L*_S_ = $\overline{AD }$= *L*_G_. When there is complete gate-source overlap and the length of the source is longer than that of the gate, the device performance will degrade. On the other hand, in the case of partial gate-source overlap, a hump effect occurs (as indicated by the circles), which can also affect the device performance. Figure 10 shows Electric band-to-band generation rate for SGO iTFET with gate-source complete overlap or partial overlap. It can be observed that the tunneling area is positively correlated with the gate-source overlap length, and that the tunneling area does not increase further when the length of the source exceeds that of the gate. In addition, in the case of partial gate-source overlap, there is tunneling not only between the gate and source, but also between the source and drain, which ultimately results in a hump effect in the device performance.

**Fig. 11:**
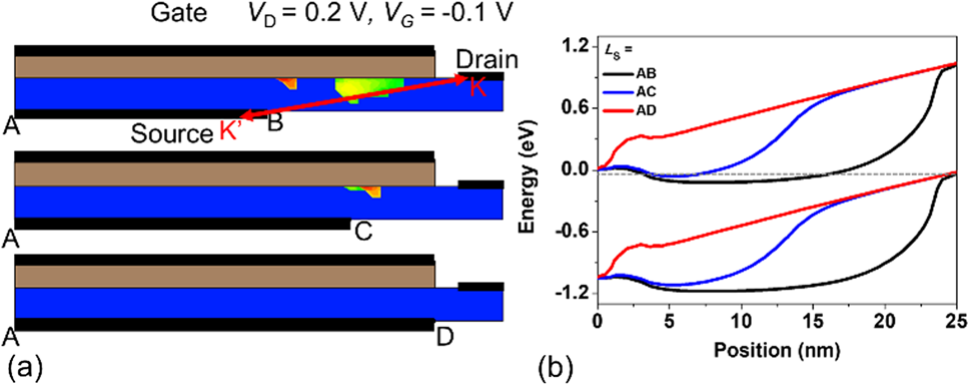
**a** Electric band-to-band generation and (b)energy band diagrams for SGO iTFET with gate-source complete overlap or partial overlap at *V*_G_ = − 0.1 V

In instances where there is partial overlap between the source and gate, the device's performance is adversely affected by both source-to-gate and source-to-drain tunneling effects. This leads to a detrimental hump in performance. It is evident from the displayed Fig. 11. (a) Electric band-to-band generation diagram, at *V*_G_ = − 0.1 V, the SGO iTFET exhibits some tunneling when the source is shorter than the gate. By analysis of Fig. 11. (b) energy band diagram along *K–K*′, we can identify that this is caused by the energy band overlapping at an oblique angle. Inconsistent control over the channel by the source gives rise to a tunneling current component, resulting in output distortion. This can be observed from the energy band diagram. Insufficient channel source control can result in oblique tunneling, which is the primary contributor to the hump effect. However, by aligning the source metal parallel to the gate metal, the control capabilities of the source and gate over the channel become consistent, and the hump effect can be eliminated. In other words, we can also explain the phenomenon by the electrical potential gradient between the gate and source-metal-region, and between the gate and non-source-metal region. Due to the presence of source metal control in one part and the absence of source metal control in another part, two sets of devices with different threshold voltages coexist. As a result, the *I*_D_–*V*_G_ characteristics exhibit a hump phenomenon. This phenomenon is similar to the appearance of parasitic edge transistors in traditional MOSFETs and involves analogous physical mechanisms.

### Schottky Barrier height (SBH) modulation

**Fig. 12:**
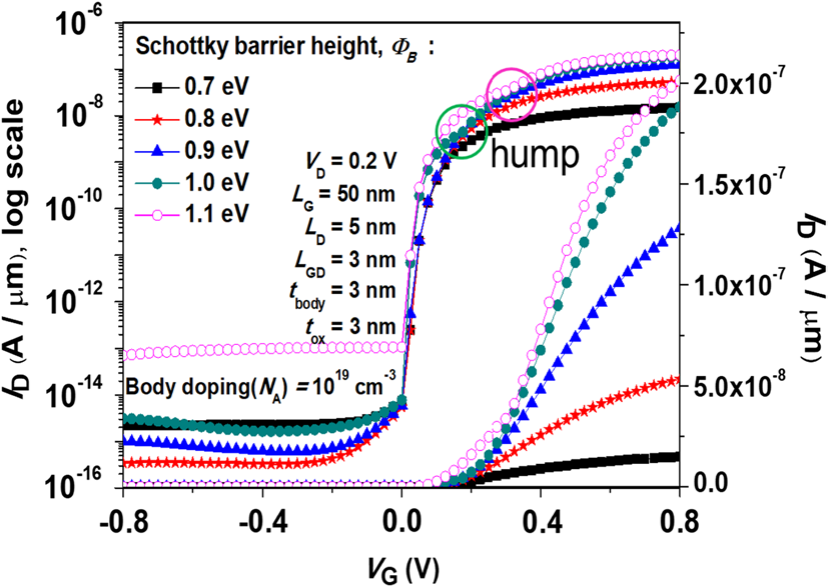
Simulated *I*_D_–*V*_G_ characteristics with various SHB for SGO iTFET. Although the on-state current increases with the increase in the SHB, a hump effect occurs at Ø_B_ ≥ 1.0 eV (as indicated by the circle), which results in a degradation of device performance

**Fig. 13:**
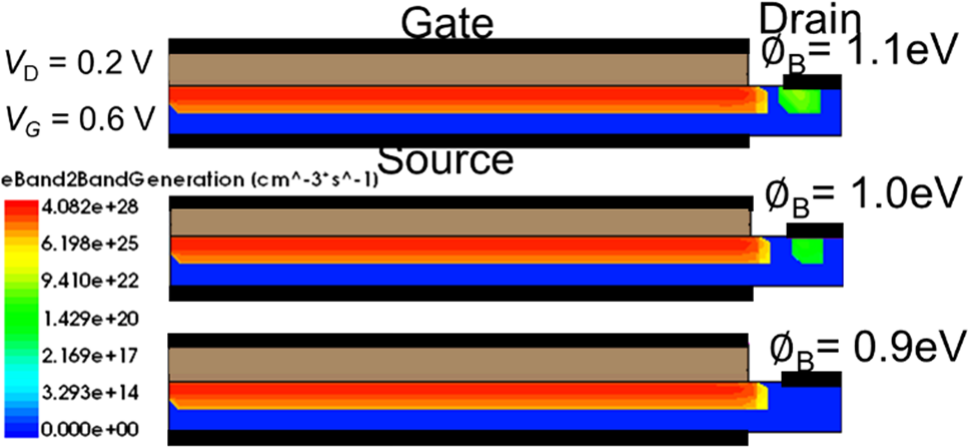
Electron band-to-band generation with different SHB. Point tunneling occurs in the drain region at Ø_B_ $\ge$ 1.0 eV compared to Ø_B_ = 0.9 eV

The following section is mainly discussed about the effect of Schottky barrier height (SBH) at the SGO iTFET. Figure 12 shows the simulated *I*_D_–*V*_G_ characteristics for SGO iTFET of various SBH. the device exhibits optimal performance at Ø_B_ = 0.9 eV. It can be observed from the figure that as the SBH increases, the *I*_ON_/*I*_OFF_ ratio significantly improves, and the device exhibits better subthreshold swing. However, a hump effect leading to a degradation of device performance occurs when the SHB exceeds 1.0 eV (as indicated by the circle). Figure 13 shows Electric band-to-band generation with different SHB. The hump effect observed at Ø_B_ = 1.0 eV is attributed to the occurrence of tunneling in the drain region. Owing to the P-type Si substrate, the establishment of a Schottky contact necessitates the fulfillment of the condition *WF*_Metal_ < *WF*_Si_. Within our device, the Schottky barrier height at the Schottky contact is quantified at 0.9 eV. Thorough calculations have discerned that a metal work function of 4.95 eV, corresponding to Tellurium (Te), aligns harmoniously with our requisites.

### Intrinsic SiGe pocket layer

**Fig. 14:**
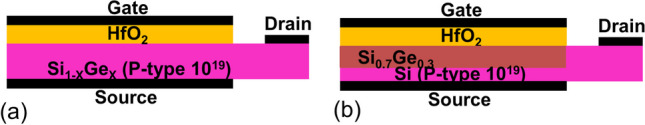
Schematics of **a** SGO iTFET (Si at *x* = 0) and **b** Pocket-SGO iTFET

**Fig. 15:**
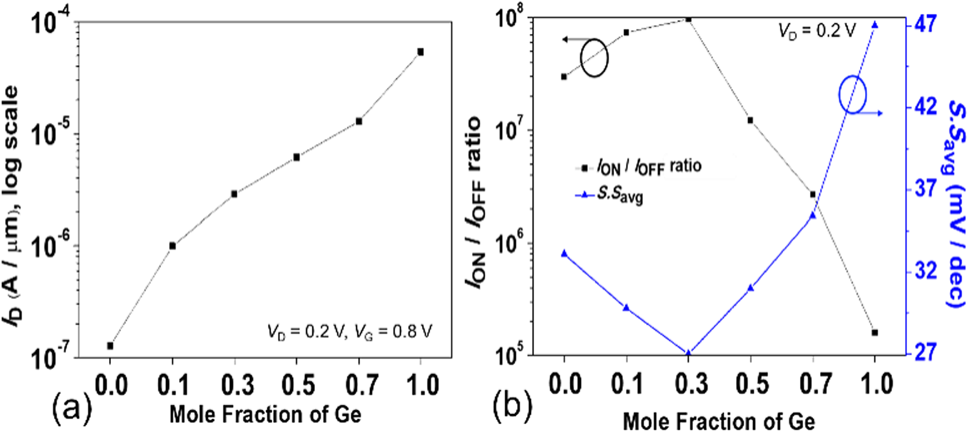
Simulated **a*** I*_ON_ current, **b** *I*_ON_/*I*_OFF_ ratio and *S.S*_avg_ for SGO iTFET with various mole fraction of Ge. Although an increase in Ge mole fraction can lead to an increase in the *I*_ON_ current, the leakage current also increases, resulting in a degradation of both *I*_ON_/*I*_OFF_ ratio and *S.S* performance

**Fig. 16:**
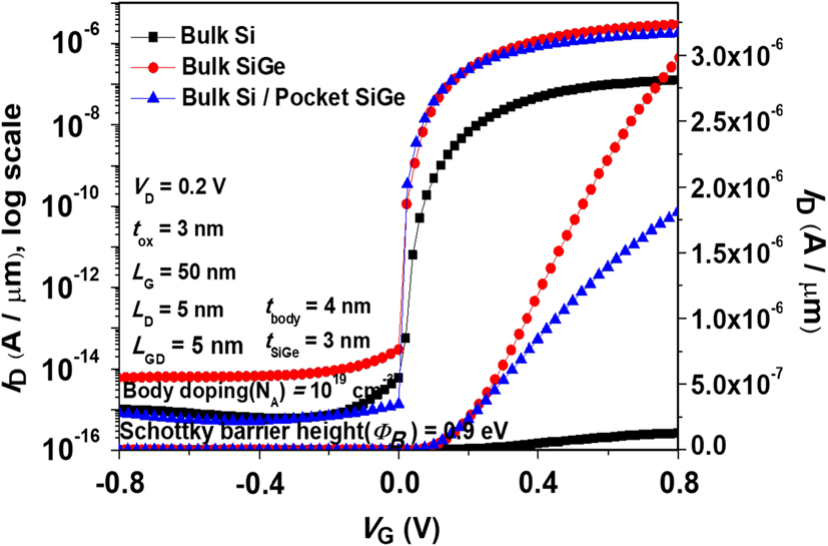
Simulated *I*_D_–*V*_G_ characteristics for SGO iTFET using different body materials. The SiGe Pocket exhibits superior *S.S* and *I*_ON_/*I*_OFF_ ratio and combines the advantages of high *I*_ON_ current of SiGe and low leakage current of Si

To further enhance the* I*_ON_ and *S.S* of SGO iTFET, we replaced the body material with narrow bandgap SiGe, as shown in Fig. 14, which shortens the band-to-band tunneling distance, increases the band-to-band tunneling probability, and improves the ON current. Figure 15 shows (a) *I*_ON_ current, (b) *I*_ON_/*I*_OFF_ ratio and *S.S*_avg_ for SGO iTFET with various mole fraction of Ge. Even though a higher Ge mole fraction can result in a higher *I*_ON_ current, it can also result in a higher leakage current, which lowers the *I*_ON_/*I*_OFF_ ratio and the *S.S* performance. Figure 16 shows the simulated *I*_D_–*V*_G_ curves for SGO iTFET with, and SiGe pocket. The SiGe Pocket has superior *S.S* and *I*_ON_/*I*_OFF_ ratio and combines the advantages of high *I*_ON_ current of SiGe and low leakage current of Si.

**Fig. 17:**
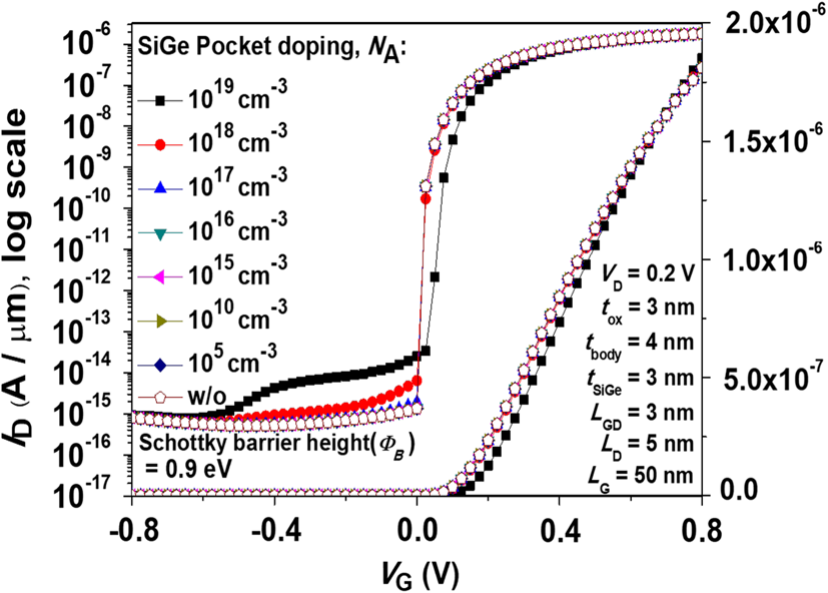
Simulated *I*_D_–*V*_G_ characteristics with various pocket concentration for Pocket-SGO iTFET. Since the device performance remains unchanged when the concentration is below 10^17^ cm^−3^, no additional ion implantation is required for the device

Furthermore, from our understanding of the effect of body concentration on device performance, we know that the doping concentration directly affects the device current and subthreshold swing. Therefore, we modulate the doping concentration of the SiGe pocket and observe its impact. Figure 17 shows the simulated *I*_D_-*V*_G_ characteristics with various pocket concentration for Pocket-SGO iTFET. It can be observed that a decrease in the doping concentration of the pocket SiGe leads to a decrease in the leakage current from 10^–13^ cm^−3^ to 10^–15^ cm^−3^. This is because a lower doping concentration of the SiGe pocket generates a larger depletion region with the body, resulting in it can effectively reduce the leakage current. Furthermore, a lower concentration also allows the* I*_ON_ current to maintain a value above 10^–5^ A/μm. Additionally, it can be observed that the device performance remains almost unchanged when the doping concentration is lower than 10^17^ cm^−3^. Considering the fabrication cost, we adopted the SiGe pocket without the need for additional ion implantation, which can significantly reduce the thermal budget compared to the conventional TFET requiring three ion implantations.

### Metal contact types of drain region

**Fig. 18:**
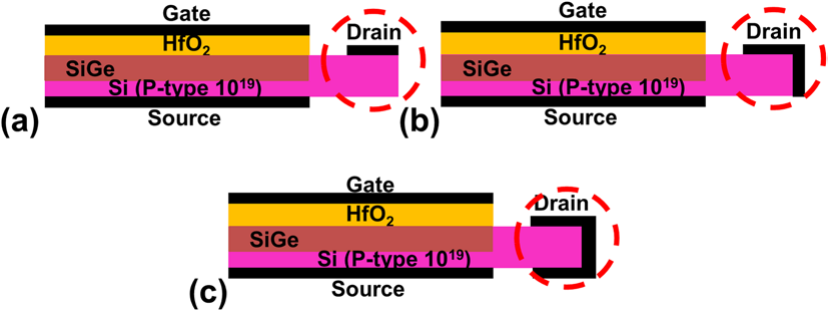
Schematics of Pocket-SGO iTFET with various contact types of drain. **a** top, **b** top and side, and **c** all around

**Fig. 19:**
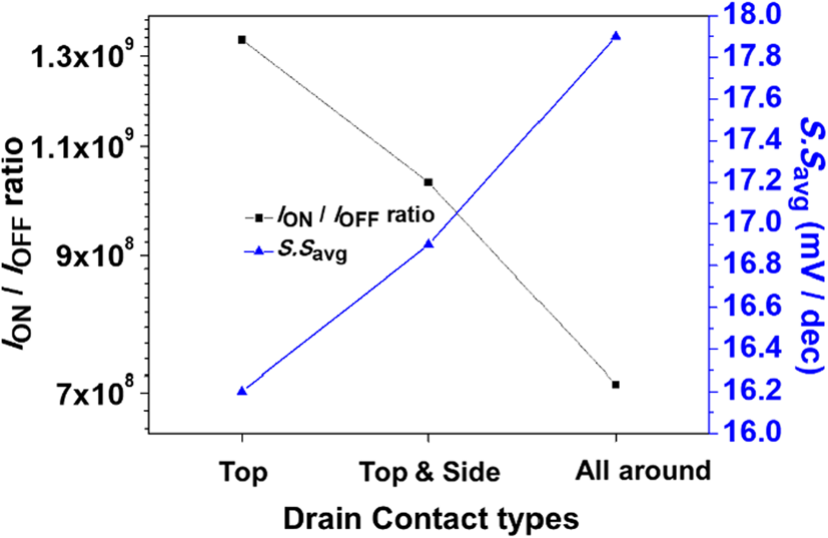
Simulated *I*_ON_/*I*_OFF_ ratio and *S.S*_avg_ for Pocket-SGO iTFET with various contact types of drain

Figure 18 shows the schematics of Pocket-SGO iTFET with various contact types of drain. (a) top, (b) top and side, and (c) all around. Increased metal contact area of the drain electrode lowers the series resistance of the device and thus increases the current. Figure 19 shows the simulated *I*_ON_/*I*_OFF_ ratio and *S.S*_avg_ for Pocket-SGO iTFET with various contact types of drain. The drain contact metal all around can make the current rise to 2.75 $\times$ 10^–5^ A/μm because of the small resistance of series connection. Although the *I*_ON_ current increases, both the *I*_ON_/*I*_OFF_ ratio and the *S.S*_avg_ degrade. After weighing the options, we chose the option with the lower *S.S* structure*,* drain metal contact at top*.*

### The issue of trap density

Since interfacial traps are unavoidable in device fabrication, we carefully considered the influence from interfacial traps between the device oxide layer and semiconductor substrate.

**Fig. 20:**
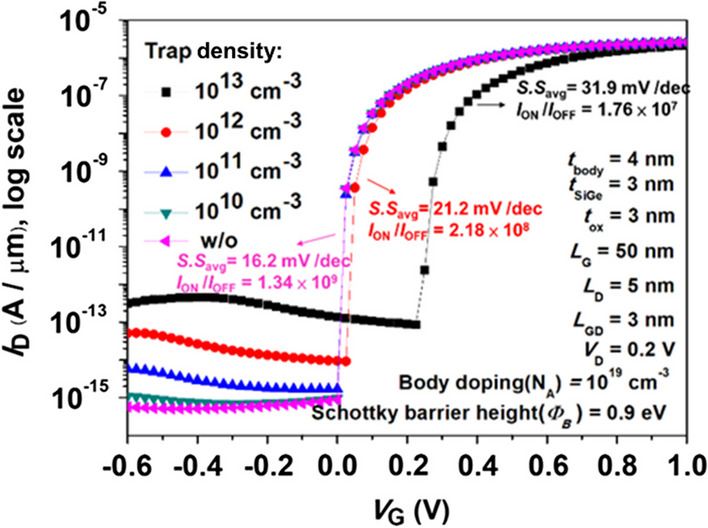
Simulated *I*_D_-*V*_G_ characteristics with various trap density for Pocket-SGO iTFET

Figure 20 shows the *I*_D_-*V*_G_ characteristics of the Pocket-SGO iTFET considering various trap density from 10^–10^ cm^−3^ to 10^–13^ cm^−3^. As the density of interfacial traps increases, the effect appears even in the off-state leakage current state where band alignment does not occur. The introduction of traps brings novel tunneling conduits for carriers across the bandgap due to trap trapping events. Therefore, the occurrence of the tunneling phenomenon leads to an upward trajectory of the leakage current curve. After considering the trap-assisted tunneling (TAT) effect, we propose that the Pocket-SGO iTFET has a specific *I*_ON_/*I*_OFF_ current ratio of 1.76 × 10^7^ and an average subthreshold swing of 31.9 mV/Dec at a trap density of 10^13^ cm^−3^. Moreover, it can be seen from the figure that the devices exhibit good *I*_ON_/*I*_OFF_ ratio and S.S. even when the trap density is lower than 10^–12^ cm^−3^. Even considering TAT, our proposed device still exhibits high reliability.

**Fig. 21:**
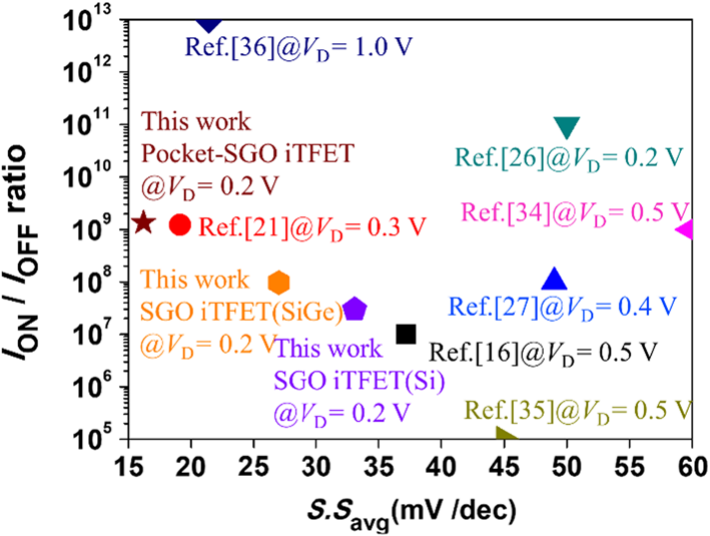
Performance comparison of low-power application devices with simulated results [16, 21, 26, 27, 34–36]

In Table 3, we do the benchmark of on-state current, average subthreshold swing, *I*_ON_/*I*_OFF_ ratio and drain voltage to compare our work with other structures. We can observe that the average subthreshold swing and *I*_ON_ of our work are relatively batter than those of other structures. It should be noted that the *V*_DS_ used for our device is 0.2 V, and the on-state current of our device is proportional to the area, *L* × *W*. As the *V*_DS_ and the area increase, the on-state current can be significantly increased accordingly. Figure 21 shows a benchmark of low-power devices proposed in recent years [16, 21, 26, 27, 34–36]. It can be concluded that our proposed device has enormous potential for development in the field of IoT and AI.

**Table 3:** Benchmark of TFET with simulated results

	Material	${I}_{\mathrm{ON}}$(A/μm)	${S.S}_{\mathrm{avg}}$(mV/Dec)	${I}_{\mathrm{ON}}/{I}_{\mathrm{OFF}}$ (A/μm)
SU TFET (2017) [16]	Si/Ge	1.35 $\times$ 10^–5^ @ *V*_D_ = 0.5 V	37.2	~ 10^7^
CG iTFET (2023) [21]	Si/SiGe	2.26 $\times$ 10^–6^ @ *V*_D_ = 0.3 V	19.12	1.22 $\times$ 10^9^
HLHSB- TFET (2023) [26]	Si	~ 10^–7^ @ *V*_D_ = 0.2 V	49	~ 10^8^
VPISDC-HSB-BTFET (2023) [27]	Si	2 $\times$ 10^–5^ @ *V*_D_ = 0.4 V	~ 50	~ 10^11^
SGO-iTFET (this work)	Si	1.28 $\times$ 10^–7^ @ *V*_D_ = 0.2 V	33.1	2.97 $\times$ 10^7^
SGO-iTFET (this work)	SiGe	2.87 $\times$ 10^–6^ @ *V*_D_ = 0.2 V	27.0	9.70 $\times$ 10^7^
Pocket-SGO iTFET (this work)	Si/SiGe	1.81 $\times$ 10^–6^ @ *V*_D_ = 0.2 V	16.2	1.34 $\times$ 10^9^

## Conclusion

In this paper, we have presented a novel Pocket-SGO iTFET design with an overlapping gate on the source, a Schottky contact to the drain, and a doping-less SiGe pocket, with the aim of achieving a steep subthreshold swing (*S.S*) and high *I*_ON_ current. By optimizing the gate and source overlap, we have significantly enhanced the tunneling efficiency while suppressing the ambipolar effect. The use of Schottky contact drain, instead of ion implantation drain, has reduced leakage current and thermal budget. Additionally, we have replaced the tunneling region with a pocket SiGe with a narrower bandgap, which has increased the probability of band-to-band tunneling and enhanced the *I*_ON_ current. Our simulations have been based on assessing the feasibility of the actual process. TCAD simulations have demonstrated that the Pocket-SGO iTFET exhibits an average and minimum subthreshold swing of *S.S*_avg_ = 16.2 mV/Dec and *S.S*_min_ = 4.62 mV/Dec, respectively. At *V*_D_ = 0.2 V, the *I*_ON_ current is 1.81 × 10^−6^ A/μm, and the *I*_ON_/*I*_OFF_ ratio is 1.34 × 10^9^. Furthermore, when considering trap-assisted tunneling, the device has still achieved S.Savg = 31.9 mV/Dec and *I*_ON_/*I*_OFF_ = 1.76 × 10^7^. The Pocket-SGO iTFET has enabled applications in IoT, large data, and low-power applications. Additionally, compared to other proposed TFETs, our architecture is simple and has a lower production cost, making it a more favorable option for circuit implementation.

## Data Availability

The corresponding author can provide the data supporting the findings of the study upon reasonable request.
